# Argument for Inclusion of Strongyloidiasis in the Australian National Notifiable Disease List

**DOI:** 10.3390/tropicalmed3020061

**Published:** 2018-06-05

**Authors:** Meruyert Beknazarova, Harriet Whiley, Jenni A. Judd, Jennifer Shield, Wendy Page, Adrian Miller, Maxine Whittaker, Kirstin Ross

**Affiliations:** 1 College of Science and Engineering, Flinders University, Bedford Park, SA 5042, Australia; harriet.whiley@flinders.edu.au (H.W.); kirstin.ross@flinders.edu.au (K.R.); 2 School of Health Medical and Applied Sciences, Centre of Indigenous Health Equity Research, Central Queensland University, Bundaberg, QLD 4670, Australia; j.judd@cqu.edu.au; 3 Department of Pharmacy and Applied Science, La Trobe University, Bendigo, VIC 3552, Australia; j.shield@latrobe.edu.au; 4 Miwatj Health Aboriginal Corporation, Nhulunbuy, NT 0881, Australia; wendy.page@my.jcu.edu.au; 5 Public Health and Tropical Medicine, James Cook University, Cairns, QLD 4870, Australia; 6 Indigenous Research Unit, Griffith University, Nathan, QLD 4111, Australia; adrian.miller@cdu.edu.au; 7 College of Public Health, Medical and Veterinary Sciences, James Cook University, Townsville, QLD 4811, Australia; maxine.whittaker@jcu.edu.au

**Keywords:** strongyloidiasis, *Strongyloides stercoralis*, notifiable, Australia

## Abstract

Strongyloidiasis is an infection caused by the helminth, *Strongyloides stercoralis*. Up to 370 million people are infected with the parasite globally, and it has remained endemic in the Indigenous Australian population for many decades. Strongyloidiasis has been also reported in other Australian populations. Ignorance of this disease has caused unnecessary costs to the government health system, and been detrimental to the Australian people’s health. This manuscript addresses the 12 criteria required for a disease to be included in the Australian National Notifiable Disease List (NNDL) under the *National Health Security Act 2007* (Commonwealth). There are six main arguments that provide compelling justification for strongyloidiasis to be made nationally notifiable and added to the Australian NNDL. These are: The disease is important to Indigenous health, and closing the health inequity gap between Indigenous and non-Indigenous Australians is a priority; a public health response is required to detect cases of strongyloidiasis and to establish the true incidence and prevalence of the disease; there is no alternative national surveillance system to gather data on the disease; there are preventive measures with high efficacy and low side effects; data collection is feasible as cases are definable by microscopy, PCR, or serological diagnostics; and achievement of the Sustainable Development Goal (SDG) # 6 on clean water and sanitation.

## 1. Introduction

Strongyloidiasis is an infection caused by the intestinal and tissue helminth, *Strongyloides stercoralis* [1]. *S. stercoralis* has been estimated to infect up to 370 million people worldwide [2]. In Australia, strongyloidiasis remains endemic in Indigenous populations, infecting communities in Queensland [3], the Northern Territory [4,5,6,7,8,9], Western Australia [10], northern South Australia [11], and northern New South Wales [12]. Seroprevalence in some communities reaches 60% [4,5,6,7,8,9,13]. Despite the prevalence and potential for morbidity and mortality posed by this disease, the true incidence in Australia remains unknown [2] as a consequence of both under-diagnosis of the disease and the absence of mechanisms to capture surveillance data [14]. The absence of reliable national data of the geographic extent and rate of transmission of this disease blinds medical and public health professionals attempting to institute effective control. This knowledge gap is not unique to Australia. Schar et al. (2013) noted that adequate information on *S. stercoralis* prevalence is still lacking from many countries, but their review found that the information that does exist points out to it being an infection that must not be neglected. They recommended information needs to be collected in a range of socio-economic and ecological settings and that in many settings the integration of control and treatment of *S. stercoralis* into a holistic helminth control program is warranted [15].

*S. stercoralis* is a soil-transmitted helminth, infecting a human when infective stage larvae penetrate the skin, enter the circulation, and subsequently travel to the lungs via the blood, from where it is swallowed into the gut [16]. This is the traditional ordered pathway, though evidence exists that random migration through the body to reach the intestine is also likely, even in primary infection [17,18,19]. A free-living phase of the parasitic life cycle occurs in the soil after host defaecation in the open, but this can only last one generation, and thus a soil reservoir of the parasite is not a factor in long-term control after implementation. Symptoms are protean, including respiratory, gastrointestinal, and skin disorders [18,20]. Unlike most other soil-transmitted helminth infections, *S. stercoralis* larvae can persist indefinitely inside the host through asexual reproduction by parthenogenesis and subsequent autoinfection [21,22].

After initial infection, there is a rapid increase in numbers as the result of an autoinfective burst [23]. This causes acute disease. This typically abates in immunocompetent people, becoming asymptomatic, or mildly symptomatic, often mimicking the symptoms of other diseases [9,24]. Due to its peculiar autoinfective nature, disease is often lifelong and a single remaining larva of *S. stercoralis* post-treatment can cause recrudescence of disease. In immunocompetent persons, the disease is chronic and long-lasting. In immunocompromised/immunosuppressed persons, or those receiving corticosteroid treatment, the infection may transform to a hyperinfective or disseminated disease syndrome, with up to 90% mortality [16,25,26,27,28,29]. 

*Strongyloides* is typically found in tropical and subtropical zones, but is mainly associated with areas of low socioeconomic status as a consequence of inadequate sanitary conditions [30]. This is supported by evidence of strongyloidiasis being found in desert communities [6,11]. Strongyloidiasis is described as the most neglected of the Neglected Tropical Diseases (NTDs) [14], and it is important to make strongyloidiasis notifiable so that epidemiological and prevalence data can be obtained to inform appropriate strategies for controlling the disease.

## 2. The Australian National Notifiable Disease Surveillance System (NNDSS)

The Quarantine Act (NSW) of 1832 was the first legislative document to cover public health issues and included mandatory reporting of diseases to local health authorities in Australia [31]. The Communicable Disease Network Australia (CDNA) was established in 1989 to enhance national communicable disease surveillance reporting to the then National Public Health Partnership (NPHP) [32]. In 2006, NPHP split into the Australian Health Protection Committee (AHPC) and the Australian Health Development Committee (AHDC). CDNA now operates under the AHPC.

The Australian National Notifiable Diseases Surveillance System (NNDSS) was first introduced in 1990 and serves as a platform to collate and report data on nationally-approved notifiable diseases from all jurisdictions to the Commonwealth [32,33]. The National Notifiable Disease List (NNDL) was created in 2008 under the National Health Security Act 2007 (Commonwealth), a document that contains a list of notifiable communicable diseases to the NNDSS. 

## 3. Criteria for Inclusion on the National Notifiable Disease List 

To determine whether a disease should be notifiable, there are currently 12 criteria against which a disease is ranked. These criteria were established by the CDNA in 2014 [32]. Table 1 presents an assessment of strongyloidiasis against each of these criteria. A score of 28 (if conservative estimates are used) to 30 (if less conservative estimates are used) was calculated for strongyloidiasis based on CDNA descriptors. The CDNA criteria state that if a disease scores less than 15, national notification is not recommended; if it falls between 15 to 25, national notification is to be considered further; and if it is higher than 25, national notification is recommended. As such, even with a conservative estimate of 28, strongyloidiasis fulfils the requirements for national notification to be recommended [32]. The criteria can be found at: (http://www.health.gov.au/internet/main/publishing.nsf/Content/8DF6148BCAC589D6CA257EE5001D0DF7/$File/Protocol-change-NNDL.pdf).

Based on Table 1 there are six key arguments for making strongyloidiasis notifiable:

- The disease is important to Indigenous health, and closing the health inequity gap between Indigenous and non-Indigenous Australians is a priority.
- A public health response is required to detect cases of strongyloidiasis and to establish the true incidence and prevalence of the disease.
- There is no alternative national surveillance system to gather data on the disease.
- There are preventive measures with high efficacy and low side effects.
- Data collection is feasible as cases are definable by microscopy, PCR, or serological diagnostics.
- Achievement of the Sustainable Development Goal (SDG) # 6 on clean water and sanitation.

## 4. Prevalence of Strongyloidiasis in Australia

Strongyloidiasis may have been present in Australia prior to the arrival of Europeans. Due to improvements in sanitation and healthcare, it is no longer typically seen in non-Indigenous Australian communities [34], but remains a major health problem for Indigenous communities, particularly in remote areas. Despite its long-term persistence in Australia, it is difficult to determine the true distribution and prevalence of the disease. The first confirmed reports of strongyloidiasis in Australia date back to the early 1900s in north Queensland [35,36,37]. The first reports of it specifically affecting Aboriginal communities (in Atherton Tablelands) date back to the early 1900s, at which time it was noted to affect Aboriginal people at almost 30 times that of non-Aboriginal [34]. To date, infection-related mortality rate in Indigenous people is much higher compared with that in non-Indigenous population. Strongyloidiasis is one of the causes of deaths [38]. Current estimates of strongyloidiasis incidence are limited and based on opportunistic testing in hotspot areas and diagnostic pathology laboratory data [39]. The former is biased towards high prevalence communities and the latter towards subjects with easy access to healthcare and laboratory services and having sufficiently symptomatic disease to require diagnostic evaluation. There is also no standard detection method available. A study conducted in north Queensland in 2006 showed a strongyloidiasis prevalence in Indigenous and non-Indigenous populations of 24% and 10% respectively [40]. An epidemiological study with Aboriginal communities in Northern Australia conducted over 2010–2011 showed a strongyloidiasis seroprevalence of 21% [7]. Overall, based on studies completed in different localized endemic areas from 1980 to 2010, strongyloidiasis prevalence is estimated to range from 2% to 41% based on faecal microscopy surveys [3,5,10,41] and from 5% to 60% based on serology survey tests [42,43]. Furthermore, infected people are known to live elsewhere in Australia apart from endemic areas [44].

Strongyloidiasis also affects other populations. GeoSentinel Surveillance Network site holds a database for returned international travelers with infectious gastrointestinal disease. Based on analysis of their international database during the period 1996–2005, *S. stercoralis* was rated the fifth most common pathogen [45]. Screening and treatment is now policy for refugees coming to Australia. However, screening has not yet been introduced to policy or systems for endemic Aboriginal communities in Australia. Immigrants and refugees from South East Asia also have high prevalence rates, as do returned travelers [20,46]. In a South Australian study, 11.6% of Vietnam veterans tested seropositive for *S. stercoralis* [47]. Four non-Indigenous cases of strongyloidiasis acquired through occupational exposure were reported in Central Australia [44].

It is difficult to estimate mortality rates associated with strongyloidiasis as it is responsible for several fatal clinical manifestations, each of which may be attributed to other causes [59]. For example, during hyperinfective strongyloidiasis, larvae migrate from the gastrointestinal system to other organs, transporting enteric bacteria with them. This can result in community-acquired septicaemia or meningitis, or local sepsis, which are then registered as cause of death on death certificates, despite the underlying cause of death being strongyloidiasis [6]. Additionally, acute strongyloidiasis can also cause severe gastrointestinal (intestinal obstruction), or respiratory (pulmonary strongyloidiasis) disease that can be potentially fatal if the strongyloidiasis is not diagnosed and treated [60,61,62].

## 5. Socioeconomic Impact Caused by Strongyloidiasis 

Due to the chronic nature of most NTDs, their burden is usually estimated using disability-adjusted life years (DALYs) lost. One DALY equals one year of life lost by a healthy person due to a disease. A study in the United States of America compared the costs and benefits of no preventive intervention and preventive intervention of 1996 people that were at risk of intestinal parasite infections, including *S. stercoralis* [54]. Preventive intervention included presumptive treatment with 400 mg of albendazole daily for five days and data was analyzed using a decision-analysis model. It was estimated that presumptive preventive intervention against human parasites would decrease DALYs by 1976 and save up to 16.4 million USD. While it is difficult to estimate treatment efficacy for an individual parasite due to complexity of the tests used in this study, strongyloidiasis was shown to cause the highest number of deaths and hospitalization costs [54]. Notably, DALYs do not describe the complete story of harmful consequences of strongyloidiasis, such as an economic impact from productivity loses, and social impact on individuals and the community [63]. DALYs are also not appropriate to use when estimating the burden of strongyloidiasis due to the underestimated prevalence and in cases of asymptomatic strongyloidiasis.

Under-diagnosis, under-treatment, and a neglected approach to this disease cause chronic strongyloidiasis cases to develop, which is costly to the health system with expensive imaging and investigations being undertaken before diagnosis. Currently, effective treatment of strongyloidiasis is available and available information technology can be used to establish a notification database. Health promotion and community engagement are also required and need to be incorporated into public health and population health strategies, which can make a difference in disease detection and treatment and ultimately, closing the gap.

## 6. Recommendation to Make Strongyloidiasis a Notifiable Disease 

Notifiable disease data are collected to estimate the prevalence of the disease, identify hotspots of infection, and determine any susceptible populations. These data will then be used by the public health institutions and authorities to implement prevention and control measures and/or interventions at local, regional, and national levels. Notification of the disease allows estimates of the effectiveness of the treatment and/or control strategies that would result in a systematic evidence-based approach to addressing this public health issue [64]. Strongyloidiasis represents a chronic, possibly widespread, potentially debilitating, and life-threatening disease endemic in Australia, which affects Indigenous communities and new Australian populations at a rate far in excess of the general population. Extra-intestinal strongyloidiasis is now listed as a notifiable disease by the Centre for Disease Control in the Northern Territory. The logical next step of national notification, and thus registration, of cases of strongyloidiasis would allow public health authorities the critical information they need to implement relevant prevention, control actions, and regulations.

Globally, it has been noted that strongyloidiasis is an underreported disease and information on at-risk and affected populations is missing [15]. This review found that information on incidence is virtually non-existent, and without this information we lack insight into ‘how often and how quickly people are re-infected after successful treatment’, ‘how often first-time infections are sustained over a longer period’, and knowledge of risks for infection for children and adults is missing. They argued for supporting longitudinal studies, especially at a community level, to address these knowledge gaps about an important infectious disease.

There is a compelling justification for strongyloidiasis to be made notifiable in order to establish prevalence data, identify the most severely affected regions and groups and subsequently implement and monitor public health interventions to control this important disease. Based on this, it is recommended that strongyloidiasis is made nationally notifiable and added to the Australian NNDL as a matter of priority.

## Figures and Tables

**Table 1:** *Strongyloides stercoralis* against 12 criteria for NNDL assessment.

#	Criterion	Score	Notes on Strongyloidiasis
Priority setting			
1	Necessity for public health response	2/4 = case reporting important for detecting outbreaks that require investigating or contacts require routine intervention	A public health response and immediate intervention is required based on the following: 1. Inadequate hygiene and sanitary conditions are the main factors for human strongyloidiasis. A person can get infected when coming into contact with or near infected human or dog faeces. In low socioeconomic status communities, such as some Indigenous communities, sanitation conditions present a high risk for strongyloidiasis transmission, contamination, re-infection, and recurrence [30]. Therefore, it is crucial to get a public health response to create and maintain adequate sanitary and hygiene conditions in the communities to prevent the disease. Culturally comprehensive health education for understanding the nature of infectious diseases and how they are transmitted is fundamental for maintaining hygienic conditions [48]. 2. There is the opportunity to highlight environmental health role in the public health response. There is an opportunity to make a difference in endemic communities and specific families/communities with high need targeting the SDG # 6 on clean water and sanitation. 3. Interventions programs such as targeted mass drug administration (MDA) have shown to be very effective in reducing the reservoir of human infection, and need to be implemented regularly on a local and national level in endemic communities [7]. 4. Another intervention program in an endemic Indigenous community incorporated *S. stercoralis* screening into the adult health check, and positive cases were treated and followed up. This selective chemotherapy intervention resulted in a decreased risk of potentially fatal hyperinfection and decreased prevalence in the community [13,49]. 5. Strongyloidiasis has been shown to prevent weight gain in children, and therefore it is critical to identify and treat *S. stercoralis* infection to avoid intervention by social services. This intervention can result in child removal from parents into care if the child shows signs of malnutrition [49]. An environmental health response to geographic hot spots would also bring in the SDG # 6 on clean water and sanitation.
2	Utility and significance of notification for prevention programs	1/4 = Need to establish burden of illness for monitoring or research purposes/priority setting	The geographic prevalence of *S. stercoralis* within Australia is essential to understand and map the hotspots. Notification and establishing the true burden of infection will improve monitoring, prevention and research, for assessing the effectiveness of prevention and control programs at the local and regional levels. Currently, there are no true disability-adjusted life years (DALYs) identified for strongyloidiasis, mainly because of poor estimates of disease prevalence.
3	Vaccine preventability	0/4 = No vaccine available	No vaccine available
4	Importance for Indigenous health	4/4 = Very high	Strongyloidiasis is endemic in the Indigenous population, affecting up to 60% of the population in some remote communities. Strongyloidiasis has been and continues to be an issue in the Australian Indigenous population, causing unnecessary morbidity and mortality in all age groups [39]. Many in the Australian Indigenous population, as a result of socioeconomic conditions and compromised/suppressed immunity due to chronic disease, are unusually susceptible to both acute strongyloidiasis, and life-threatening disseminated and/or hyperinfective strongyloidiasis.
5	Emerging or re-emerging disease	2/4 = slowly re-emerging or increasing incidence/prevalence disease over the past 5 years	Strongyloidiasis has been called ‘the most neglected of Neglected Tropical Diseases’ [14]. Cases have been reported since the early 1900s. The literature shows that the prevalence of the disease trend declined following mass drug administration (MDA) of ivermectin (2010) and albendazole (1995) in these communities [7,13]. However, the disease has never been eliminated and tends to reappear [5,41]. The disease has been neglected, and the real prevalence of the disease is underestimated due to lack of disease surveillance. Due to the unique autoinfective cycle of *S. stercoralis*, chronic strongyloidiasis lasts for a lifetime if not effectively diagnosed and treated. Cases of hyperinfection and iatrogenic fatal dissemination are predicted to increase as the infected populations age and are at a higher risk of being immunosuppressed. Corticosteroids have been considered a factor in 65% of fatalities from hyperinfection [50]. Another factor contributing to this emerging disease status with increasing cases of severe, complicated strongyloidiasis, has been the lack of awareness of strongyloidiasis in medical personnel who have been trained in Australia.
6	Communicability and potential for outbreaks	2/4 = Medium	There is a potential for outbreaks in poor-infrastructure settings with low sanitary and hygiene conditions, which together produce a high risk for strongyloidiasis transmission from person to person via faecal-skin and faecal-oral routes [51].
7	Severity and socioeconomic impacts	1/4 = low severity and socioeconomic impacts in chronic strongyloidiasis (strongyloidiasis in healthy person) or 2/4 = medium severity and socioeconomic impacts in disseminated or hyperinfective strongyloidiasis	In healthy people, chronic strongyloidiasis may have only mild, intermittent, and non-specific symptoms. However, the autoinfection feature of this helminth and parthenogenesis, allows single larvae reproducing within the host leading to a chronic, long-lasting disease. If not diagnosed and treated, the disease can take a more serious form as the person becomes immunocompromised/immunosuppressed, with an often-fatal outcome. A case fatality rate of almost 90% has been reported [6]. Strongyloidiasis presents unnecessary cost to the health systems, as strongyloidiasis is both preventable and treatable if diagnosed early, and in the chronic stage. The diagnostic and treatment costs, including selective chemotherapy, targeted MDA and water, sanitation and hygiene (WASH) have been estimated in previous research and shown to be affordable [52,53]. It was estimated in US citizens that presumptive preventive intervention would decrease DALYs caused by intestinal parasites, including *Strongyloides*, by up to 1976- saving USD 16.4 million [54].
8	Preventability	4/4 = preventive measure with high efficacy/low side effects/high acceptability and uptake	Adequate sanitary and hygiene conditions including safe water supply, proper toileting and hygiene facilities would provide long term sustainable prevention and elimination of strongyloidiasis [51]. This should be combined with health education and research to determine the gold standard for strongyloidiasis diagnosis. Treatment of chronic strongyloidiasis prevents hyperinfection. Currently, ivermectin is the drug of first choice to treat human strongyloidiasis, followed by albendazole [55]. Ivermectin and albendazole, given according to therapeutic guidelines for strongyloidiasis [41], have been shown to eliminate the disease in 70% to 85% of those with chronic strongyloidiasis. Both drugs have negligible side effects. Ivermectin requires only one to two administrations. Albendazole requires two courses of daily doses for three days. A single dose is ineffective [7,13].
9	Level of public concern and/or political interest	2/4 = low to medium public concern or political interest or 3/4 = medium to high public concern or political interest	Strongyloidiasis is an overlooked, neglected disease [14]. However, when people are made aware of the disease, there is high public concern. This is illustrated by a recently published article on strongyloidiasis in ‘The Conversation’ which received a large number of responses by the general public showing their interest and concern about the disease [56]. Closing the Gap (the health inequity gap between Indigenous and non-Indigenous Australians) is a high priority in mainstream Australia [57]. The fact that locally-acquired infection in Australia is almost exclusively seen in Indigenous communities should be of great public and political concern.
Feasibility of collection			
10	A case is definable	4/4 = Case has an acceptable laboratory definition with or without a clinical definition	A strongyloidiasis case is definable and we propose to notify strongyloidiasis by the laboratories based on positive serology or parasitological diagnosis [58]. In disseminated and hyperinfective strongyloidiasis, faecal examination has higher sensitivity due to large numbers of viable larvae and the patient is usually in a hospital setting at the time of diagnosis. In immunocompetent persons, chronic strongyloidiasis might not always be detected by microscopy due to low and irregular larval load, and serology has the highest sensitivity and is recommended [29].
11	Data completeness is likely to be acceptable	2/4 = Data represent a proportion of community cases with a known undercount	Data on the prevalence of strongyloidiasis is limited. Studies suggest that up to 60% of the population in Indigenous rural or remote communities is infected with strongyloidiasis. A study in North Queensland found that 10% of the non-Indigenous population has strongyloidiasis [40]. It is believed that the disease is likely to be more widespread in Australia that the current data suggest.
12	Alternative surveillance mechanisms	4/4 = No alternative surveillance mechanisms in place.	There is no surveillance mechanism available to monitor and report on strongyloidiasis.

Total score: 28–30.

## Dedication

The authors would like to dedicate this work to Emeritus Professor Rick Speare. He spent much of his life understanding *S. stercoralis*, preventing and managing strongyloidiasis in Indigenous communities. He argued for inclusion of strongyloidiasis to the Australian National Notifiable Disease List as means of controlling the disease.
